# Non-coding RNA *ANRIL* and the number of plexiform neurofibromas in patients with *NF1* microdeletions

**DOI:** 10.1186/1471-2350-13-98

**Published:** 2012-10-26

**Authors:** Tanja Mußotter, Lan Kluwe, Josef Högel, Rosa Nguyen, David N Cooper, Victor-Felix Mautner, Hildegard Kehrer-Sawatzki

**Affiliations:** 1 Institute of Human Genetics, University of Ulm, Albert-Einstein-Allee 11, Ulm, Germany; 2 Department of Maxillofacial Surgery, University Medical Center Hamburg Eppendorf, Hamburg, Germany; 3 Department of Neurology, University Hospital Hamburg Eppendorf, Hamburg, Germany; 4 Institute of Medical Genetics, School of Medicine, Cardiff University, Cardiff, UK

## Abstract

**Background:**

Neurofibromatosis type-1 (NF1) is caused by mutations of the *NF1* gene at 17q11.2. In 95% of non-founder NF1 patients, *NF1* mutations are identifiable by means of a comprehensive mutation analysis. 5-10% of these patients harbour microdeletions encompassing the *NF1* gene and its flanking regions. NF1 is characterised by tumours of the peripheral nerve sheaths, the pathognomonic neurofibromas. Considerable inter- and intra-familial variation in expressivity of the disease has been observed which is influenced by genetic modifiers unrelated to the constitutional *NF1* mutation. The number of plexiform neurofibromas (PNF) in NF1 patients is a highly heritable genetic trait. Recently, SNP rs2151280 located within the non-coding RNA gene *ANRIL* at 9p21.3, was identified as being strongly associated with PNF number in a family-based association study. The T-allele of rs2151280, which correlates with reduced *ANRIL* expression, appears to be associated with higher PNF number. *ANRIL* directly binds to the SUZ12 protein, an essential component of polycomb repressive complex 2, and is required for SUZ12 occupancy of the *CDKN2A/CDKN2B* tumour suppressor genes as well as for their epigenetic silencing.

**Methods:**

Here, we explored a potential association of PNF number and PNF volume with SNP rs2151280 in 29 patients with constitutional *NF1* microdeletions using the exact Cochran-Armitage test for trends and the exact Mann–Whitney–Wilcoxon test. Both the PNF number and total tumour volume in these 29 NF1 patients were assessed by whole-body MRI. The *NF1* microdeletions observed in these 29 patients encompassed the *NF1* gene as well as its flanking regions, including the *SUZ12* gene.

**Results:**

In the 29 microdeletion patients investigated, neither the PNF number nor PNF volume was found to be associated with the T-allele of rs2151280.

**Conclusion:**

Our findings imply that, at least in patients with *NF1* microdeletions, PNF susceptibility is not associated with rs2151280. Although somatic inactivation of the *NF1* wild-type allele is considered to be the PNF-initiating event in NF1 patients with intragenic mutations and patients with *NF1* microdeletions, both patient groups may differ with regard to tumour progression because of the heterozygous constitutional deletion of *SUZ12* present only in patients with *NF1* microdeletions.

## Background

Neurofibromatosis type 1 (NF1; MIM# 613113) is an autosomal dominant inherited disease, with an incidence of 1 in 3000, caused by mutations of the *NF1* gene at 17q11.2. In 95% of non-founder NF1 patients, *NF1* gene mutations are identified when a comprehensive *NF1* mutation analysis is applied, including an RNA-based core assay supplemented with methods to identify *NF1* microdeletions [1]. The proportion of patients with large deletions (termed *NF1* microdeletions) that encompass the entire *NF1* gene and its flanking regions among all patients with NF1 is 5–10 % [2].

NF1 is a tumour predisposition syndrome characterised by tumours of the peripheral nerve sheaths including the pathognomonic neurofibromas. Cutaneous or dermal neurofibromas (DNF) usually grow during puberty or early adulthood at the end of single peripheral nerves and form small round tumours on the skin which never become malignant. In contrast to DNF, plexiform neurofibromas (PNF) grow along large nerve trunks involving several nerve bundles and mostly represent much larger and more complex tumours than DNF. PNF are usually congenital [3], can grow continuously and may cause organ compression, neurologic impairment and motor dysfunction. At least 10% of all PNF transform into malignant peripheral nerve sheath tumours (MPNST) which are the major cause of NF1-associated mortality [4].

**Figure 1:**
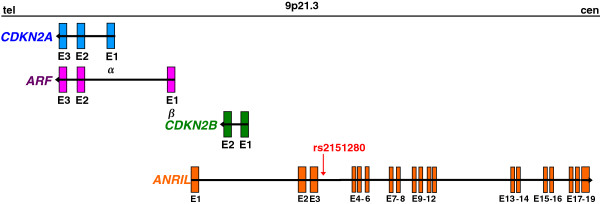
**Schematic representation of the genomic organization of the *CDKN2A/*ARF*-CDKN2B *gene cluster on chromosome 9p21.3.***CDKN2A* and ARF share the same exons 2 and 3 but include different first exons (1α and 1β). The alternative splicing of exon 1α or exon 1β to exons 2 and 3 gives rise to a difference in the reading frames between *CDKN2A* and ARF, thereby accounting for the absence of any amino acid homology between the proteins encoded by *CDKN2A* and ARF. The long non-coding RNA *ANRIL* (antisense non-coding RNA in the *INK4* in locus) gene contains 19 exons and is transcribed in the antisense orientation of the *CDKN2A/*ARF*-CDKN2B* gene cluster. Black arrows indicate the transcriptional orientation of the genes at 9p21.3. The red arrow indicates the relative position of the SNP rs2151280 within *ANRIL* intron 3.

NF1 is associated with considerable inter- and intra-familial variability in phenotypic expression. Nevertheless, the familial aggregation of specific symptoms suggests the influence of a strong genetic component unrelated to the constitutional *NF1* mutation [5,6]. One of the phenotypic traits with the highest estimated heritability in NF1 is the number of PNF, suggesting that one or more modifier genes might influence PNF susceptibility [6]. Recently, a single nucleotide polymorphism (SNP) rs2151280, located within the non-coding RNA gene *ANRIL* at 9p21.3, has been identified as being associated with the number of PNF in a family-based association study [7]. *ANRIL* (antisense non-coding RNA in the *INK4*locus) is transcribed in the antisense orientation to the *CDKN2A/*ARF and *CDKN2B* genes (Figure 1) and is known to influence their expression [8-10]. *CDKN2A/*ARF and *CDKN2B* are three tumour suppressor genes which play a central role in cell cycle inhibition, senescence and stress-induced apoptosis [11]. Importantly, homozygous deletion or expression silencing of these genes has been observed in a subset of PNF, atypical neurofibromas (considered as premalignant tumours) and MPNSTs indicative of their role during the malignant progression of peripheral nerve sheath tumours [7,12,13]. However, not only the malignant progression of PNF but also their formation may be influenced by genes at 9p21.3. This conclusion has been drawn from the observed association between the number of PNF in NF1 families and SNP rs2151280 located within the *ANRIL* gene. The T-allele of rs2151280 has been found to be associated with a higher number of PNF [7]. These authors investigated a total of 1105 individuals (740 NF1 patients and 365 unaffected relatives from 306 French NF1 families). It is however unclear how the number of PNF was assessed in these 740 NF1 patients. Whilst PNF can be externally visible tumours, they may also present as internal asymptomatic tumours which are not detectable by physical examination. Hence, the accurate and reliable detection of all PNF in a given patient with NF1 requires whole-body magnetic resonance imaging (MRI) [14]. In this study, we analysed 29 patients with non-mosaic *NF1* microdeletions. The number of PNF as well as the total PNF volume exhibited by these patients has been thoroughly analysed by whole-body MRI and volumetric analysis in a previous study [15]. The 29 patients with *NF1* microdeletions analysed here presented with different numbers of PNF (ranging from 0 to 5 PNF per patient). The purpose of our study was to examine if there was an association between *ANRIL* SNP rs2151280 and the number or volume of PNF observed in patients with *NF1* microdeletions.

## Methods

### Patients and number or volume of PNF

All 29 NF1 patients (or their parents) investigated gave their written informed consent for the studies to be performed. This study was approved by the university ethics committees of the participating institutions. The patients harboured non-mosaic (i.e. constitutional) *NF1* microdeletions (Table 1). The extent of these deletions, as well as the absence of normal cells without the deletion, has already been determined by FISH and sequence analysis of the breakpoints using DNA derived from peripheral blood of the patients as described previously [16-21].

**Table 1:** **Number of plexiform neurofibromas (PNF), tumour volume and genotype of SNP rs2151280 in 29 patients with non-mosaic *NF1 *microdeletions**

**Patient**	**Age (years)**	**Sex**	**Total tumour volume (ml)**	**Relative tumour volume (ml/kg**^**a**^**)**	**Number of PNF**^**b**^	**Extent (type) of*NF1*microdeletion**	**Genotype of rs2151280**
682	53	female	0	0	0	1.4-Mb (1)^c^	C/T
1058	4	female	0	0	0	1.4-Mb (1)^c^	C/T
748	21	female	0	0	0	1.4-Mb (1)^c^	C/C
876	15	female	0	0	0	1.4-Mb (1)^c^	T/T
975	11	male	0	0	0	1.4-Mb (1)^c^	C/C
991	18	male	0	0	0	1.4-Mb (1)^c^	C/T
807	18	female	0	0	0	1.4-Mb (1)^c^	C/C
284	14	female	0	0	0	1.4-Mb (1)^c^	C/T
1053	28	female	1889	30.5	1	1.4-Mb (1)^c^	C/C
272	16	female	258	4.8	1	3-Mb (atypical)^d^	C/C
321	23	male	54	0.6	1	1.4-Mb (1)^c^	C/C
793	45	male	39	0.43	1	1.4-Mb (1)^c^	C/C
82	20	female	28	0.35	1	2.7-Mb (atypical)^e^	T/T
515	39	female	13	0.15	1	1.4-Mb (1)^c^	C/T
978	9	female	10	0.33	1	1.4-Mb (1)^c^	C/T
917	31	female	8	0.12	1	1.4-Mb (1)^c^	C/C
283	40	female	463	6.4	2	1.4-Mb (1)^c^	C/T
726	28	female	215	2.05	2	1.4-Mb (1)^c^	T/T
941	15	male	20	0.28	2	1.4-Mb (1)^c^	C/T
463	26	male	5677	77.8	3	2-Mb (atypical)^f^	C/T
1056	38	male	4986	59.4	3	1.4-Mb (1)^c^	C/T
928	28	female	468	9.0	3	1.3-Mb (atypical)^g^	C/T
714	26	female	321	5.35	3	1.4-Mb (1)^c^	C/C
579	17	male	8018	114.5	4	1.4-Mb (1)^c^	C/T
1057	17	male	3896	62.8	4	1.2-Mb (2)^h^	C/T
33	24	male	724	9.3	4	1.4-Mb (1)^c^	C/C
857	3	female	656	29.8	4	1.4-Mb (1)^c^	C/T
866	16	female	669	10.6	5	1.4-Mb (1)^c^	C/T
1059	22	male	247	3.43	5	1.4-Mb (1)^c^	C/T

a: Tumour volume relative to body weight of the patient on the MRI scan date.

b: The plexiform neurofibromas (PNF) counted were > 3 cm in diameter.

c: as reported in [16], d: as reported in [17], e: published in [18]; f: published in [19]; g: as published in [20]; h: as published in [21].

The number of plexiform neurofibromas (PNF) and the total tumour volume exhibited by the 29 patients with *NF1* microdeletions have been investigated in our previous study by whole-body magnetic resonance imaging (MRI) and subsequent volumetric evaluation of the tumours [15]. In brief, the entire body of each patient was investigated in a series of 10 mm slices performed by the 1.5 Tesla Siemens Magnetom 63 SP/Symphony/Avanto scanner (Siemens, Munich, Germany). PNF were detected based on their characteristic appearance as signal-intense lesions >3 cm in their longest diameter. The tumour volume was calculated using MedX software (v3.42; Sensor Systems, Inc, Sterling, Virginia) as previously described [14]. PNF are often complex tumours that grow along nerves and the extent of each tumour was carefully evaluated by the analysis of multiple subsequent images.

### Genotype of SNP rs2151280

The genotype of the single nucleotide polymorphism (SNP) rs2151280, located within intron 3 of the *ANRIL* gene, was investigated by PCR amplification of the respective genomic region and subsequent sequence analysis of PCR fragments amplified from blood-derived DNA of the 29 patients with *NF1* microdeletions. PCRs were performed with primer 5′ GGGAAGAGATGAAGTAGTCAATAAAA 3′ and primer 5′ CCCTCAGCAGCACTTATTTTC 3′. DNA was extracted from whole blood using the DNeasy Blood and Tissue Kit (Qiagen, Hilden, Germany).

### Statistical analysis

In order to explore the potential association between the number of PNF in the 29 patients with *NF1* microdeletions and the genotype of SNP rs2151280, we used two different statistical tests, the exact Cochran-Armitage test for trends and the exact Mann–Whitney–Wilcoxon test. To assess the extent of any association between the tumour volume and rs2151280, the exact Mann–Whitney–Wilcoxon test was applied. An adjustment for age was performed by means of a generalized linear model with a negative binomial distribution and a log-link function. Correlations between the number of PNF, the tumour volume observed and the age of the patients were tested for by determination of the Spearman rank correlation coefficient. Statistical analyses were performed using Statistical Analysis System (SAS) software.

## Results

In the present study, we investigated the potential association between the T-allele of SNP rs2151280 and both the number and volume of the PNF exhibited by 29 patients with non-mosaic *NF1* microdeletions. These patients harboured a total of 52 PNF > 3 cm as determined previously by whole-body magnetic resonance imaging (MRI) [15]. Tumours smaller than 3 cm in diameter were not included in the analysis because these tumours cannot be evaluated by volumetric analysis. At least 13 (25%) of 52 tumours analysed were not visible by external physical examination but were detected exclusively by MRI.

We used the Cochran-Armitage test for trends to investigate a potential association between the number of PNF observed in the 29 patients with *NF1* microdeletions and SNP rs2151280 . By means of this test, an association between variable (a) with two categories and variable (b) with >2 categories was investigated. In the context of this study, variable (a) represents the genotype of SNP rs2151280 observed in the 29 patients. Variable (a) has been classified into two categories: category #1 represents the genotypes homozygous for the C-allele whereas category #2 represents the CT and TT genotypes of SNP rs2151280. Variable (b) represents the number of PNF observed, with six distinct categories being classified, ranging from category #1 (no PNF), category #2 (one PNF), category #3 (two PNF) etc. up to category #6 (five PNF) (Table 1). Within each category of variable (b), the frequency of categories #1 and #2 of variable (a) was investigated. In other words, all 29 patients were classified into categories depending upon the number of PNF observed. Subsequently, the frequency of the CC genotype vs. the CT and TT genotypes was assessed for each category of patient distinguishable by PNF number. A significant association between the PNF number and the frequency of the CC vs. CT/TT genotypes was not observed (*P* = 0.20, exact Cochran-Armitage test for trends).

The number of PNF in the 10 patients with a CC genotype ranged from 0 to 4 tumours with a mean value of 1.2 PNF per patient. By contrast, in the 19 patients with the genotype CT or TT, the number of PNF ranged from 0 to 5 with a mean value of 2.1. However, the observed difference between these groups of patients (those with a CC genotype and those with a CT or TT genotype) did not attain statistical significance (*P* = 0.21, exact Mann-Whitney-Wilcoxon test). Although PNF are mostly congenital tumours and hence the age of the patients investigated is not considered to be critical, we included an adjustment for age in our comparisons. Again, the difference in the PNF number observed in both patient groups was not found to be significant (*P* = 0.16, with an adjustment for age).

We also investigated a putative association between the tumour volume normalized against body weight and the rs2151280 genotype (CC vs. CT and TT genotypes) in the 29 *NF1* microdeletion patients. In the group of patients with the CC genotype, the mean tumour vol-ume was 5.1 ml/kg whereas the median tumour volume was 0.52 ml/kg (95% CI: 0.00 - 9.30). In the 19 patients with CT or TT genotypes, the mean and median tumour volume were 19.8 ml/kg and 2.05 ml/kg (95% CI: 0.15 - 29.80), respectively. Although both groups of patients differed considering the median tumour volume, the confidence intervals overlap to a large extend. A significant difference in tumour volume was not detected comparing both groups of patients (*P* = 0.52, exact Mann–Whitney–Wilcoxon test; *P* = 0.19 with adjustment for age). We also did not observe a significant correlation between the total tumour volume (not normalized against body weight) or the number of PNF and the age of patients (*ρ* = 0.22; *P* < 0.24; *ρ* = 0.11; *P* < 0.58). By contrast, a correlation between the total tumour volume and the number of tumours was observed (*ρ* = 0.87; *P* < 0.001).

## Discussion

The chromosome 9p21.3 region harbours a cluster of important growth regulatory genes (*CDKN2A/*ARF and *CDKN2B)* that are deleted or transcriptionally silenced in a wide range of tumours such as plexiform neurofibromas (PNF) [12,22]. The proteins encoded by the *CDKN2A/CDKN2B* genes act as inhibitors of the CDK4/6 cyclin-dependent kinases, thereby regulating the growth suppressive activity of the RB family of proteins. By contrast, the ARF protein binds to and inhibits the oncoprotein MDM2 which activates p53 [11]. The expression of *CDKN2A*, ARF and *CDKN2B* is very low in both young and non-neoplastic cells but increases during cell aging and oncogene-induced hyperproliferation, suggesting that the coordinated expression of these genes is a means to regulate senescence and prevent oncogene-driven hyperproliferation [11]. The polycomb repressive complexes PRC1 and PRC2 have been shown to initiate and maintain the silenced state of the *CDKN2A/*ARF, *CDKN2B* gene cluster [23,24]. PRC1 and PRC2 are recruited to these loci by the 3.8-kb non-coding RNA *ANRIL* in order to regulate their expression [8,10,25,26].

In a family-based association study, Pasmant et al. [7] investigated a total of five tag SNPs located at 9p21.3 in 1105 individuals (740 NF1 patients and 365 unaffected individuals from 306 NF1 families) and observed a significant association between the number of PNF and one of these five SNPs, rs2151280. This SNP, located within intron 3 of the *ANRIL* gene, was found to be associated with the number of PNF under a dominant model, with preferential transmission of the derived T-allele to those NF1 patients possessing a higher number of PNF. By contrast, the number of dermal neurofibromas (DNF) was not found to be associated with rs2151280. Importantly, the T-allele of rs2151280 is associated with a reduced *ANRIL* expression level suggesting either a functional role for SNP rs2151280 or that this SNP is in linkage disequilibrium with an additional as yet unknown functional variant which influences *ANRIL* expression [7]. Taken together, these findings suggested that modulation of *ANRIL* expression mediates PNF susceptibility in patients with NF1. It is unclear how many patients with *NF1* microdeletions were included in the study of Pasmant et al. [7]. However, only 5% of patients with NF1 exhibit *NF1* microdeletions and familial cases are very rare.

In this study, we investigated a putative association between the number or volume of PNF and rs2151280 in 29 patients with non-mosaic *NF1* microdeletions. These patients were extremely well characterized by whole-body MRI. We did not observe an association between the T-allele of rs2151280 and either PNF number or PNF volume in these patients, suggesting that this SNP does not exert a strong effect on PNF susceptibility in this group of *NF1* microdeletion patients. However, we cannot rule out the possibility of a weak association that might have remained undetected owing to the small number of patients investigated. Under the assumption of an ordered categorical distribution, we estimated that it would have been necessary to analyze approximately 300 NF1 patients to detect a significant association between tumour volume and the T-allele with a power of 80% using the Mann–Whitney–Wilcoxon test (α=5%). This estimation is however based on the observations we made in the 29 patients and implies that the distribution of tumour volumes observed is representative for the whole population of *NF1* microdeletion patients. Since *NF1* microdeletions are rare (occurring with an estimated prevalence of 1:70,000), the whole-body MRI investigation of 300 patients with *NF1* microdeletions is scarcely feasible. As deduced from the data obtained from the analysis of the 29 *NF1* microdeletion patients, a strong association between the T-allele of SNP rs2151280 and the PNF load is not obvious.

Patients with *NF1* microdeletions have been reported to exhibit a more severe clinical phenotype than patients with intragenic *NF1* mutations, as evidenced by an increased risk of MPNSTs, severe learning disability, cognitive impairment, developmental delay and dysmorphic facial features [16,27-30]. However, the number of PNF, as determined by whole-body MRI, was not found to differ significantly between patients with *NF1* microdeletions as a group and NF1 patients lacking large *NF1* deletions [15]. Nevertheless, differences in PNF development and biology may well exist between both patient groups i.e. those with *NF1* microdeletions and those with intragenic *NF1* mutations. The most common type of *NF1* microdeletion encompasses 1.4-Mb (termed type-1 *NF1* deletion) and is associated with the loss of 14 protein-coding genes inclusive of the *NF1* gene [31-33]. Potentially, the loss of one or several of the genes located within the *NF1* microdeletion region in addition to the deletion of the *NF1* gene, may influence tumour biology or progression. A good candidate for such a modifier gene influencing tumour development is *SUZ12* which is located within the 1.4-Mb *NF1* microdeletion region. One allele of *SUZ12* is deleted in all patients investigated in our study. The SUZ12 protein is an essential component of the polycomb repressive complex 2 (PRC2) and somatic mutations as well as deletions of *SUZ12* have recently been identified in various haematological malignancies indicating an important role for chromatin modifiers (such as the polycomb repressive complexes) in tumorigenesis [34-39]. Remarkably, the polycomb repressive complexes 1 and 2 (PRC1 and PRC2) have also been shown to regulate the expression of the *CDKN2A/*ARF and *CDKN2B* genes. *ANRIL* directly binds to SUZ12, an essential component of PRC2 and is required for SUZ12 occupancy of the *CDKN2B* locus as well as for the epigenetic silencing of *CDKN2B*[8].

The loss of one *SUZ12* allele in patients with germline *NF1* microdeletions may well influence *ANRIL-*mediated expression regulation of the *CDKN2A/CDKN2B* tumour suppressor genes. Although somatic inactivation of the *NF1* wild-type allele is considered to be the PNF-initiating event in NF1 patients with intragenic mutations and patients with *NF1* microdeletions [40], both patient groups may differ with regard to tumour progression because of the heterozygous constitutional deletion of *SUZ12* present only in patients with *NF1* microdeletions. Consistent with this hypothesis, an extremely high total PNF volume (>3,000 ml) was noted significantly more frequently in patients with *NF1* microdeletions than in NF1 patients without large deletions [15].

## Conclusions

Our findings in the present study suggest that the putative modulation of *ANRIL* expression by the T-allele of SNP rs2151280 does not influence PNF susceptibility in patients with *NF1* microdeletions. Further studies are however needed in order to investigate possible differences in PNF development or susceptibility in NF1 patients with and without *NF1* microdeletions.

## Abbreviations

ANRIL: Antisense non-coding RNA in the *INK4*locus; CDK4/6: Cyclin-dependent kinases 4 and 6; CDKN2A: Cyclin-dependent kinase inhibitor 2A; DNF: Dermal neurofibroma; MDM2: Mouse double minute 2 homologue; MPNST: Malignant peripheral nerve sheath tumour; NF1: Neurofibromatosis type-1; PNF: Plexiform neurofibroma; SUZ12: Suppressor of zeste homolog 12; ARF: Alternate open reading frame.

## Competing interests

The authors declare that they have no competing interests.

## Authors’ contributions

TM carried out the molecular studies and drafted the manuscript. LK, VFM and RN performed the clinical investigations of the patients. JH performed the statistical analysis. HKS and DNC participated in the study design and wrote the manuscript. All authors read and approved the final manuscript.

## Pre-publication history

The pre-publication history for this paper can be accessed here:

http://www.biomedcentral.com/1471-2350/13/98/prepub
